# Age-Dependent Labor Division and Behavioral Characteristics in *Vespa velutina nigrithorax* (Hymenoptera: Vespidae) Under Laboratory Conditions

**DOI:** 10.3390/insects17090943

**Published:** 2026-09-09

**Authors:** Dongeui Hong, Gard W. Otis, Chuleui Jung

**Affiliations:** 1 Department of Plant Medicals, Gyeongkuk National University, Andong 36729, Republic of Korea; ehd2678@naver.com; 2 School of Environmental Sciences, University of Guelph, Guelph, ON N1G 2W1, Canada; gotis@uoguelph.ca; 3 Institute of Bee Health, University of Bern, CH-3097 Bern, Switzerland; 4 Agricultural Research Institute, Gyeongkuk National University, Andong 36729, Republic of Korea

**Keywords:** temporal polyethism, eusocial insects, task transition, colony dynamics, worker behavior

## Abstract

Eusocial insects maintain their colonies through diverse cooperative tasks, yet patterns among these behaviors remain poorly understood in hornets. To examine temporal polyethism, we observed a *Vespa velutina nigrithorax* colony under controlled laboratory conditions (26 °C, 60% RH). Workers were categorized into 5-day cohorts and monitored across five tasks: nest hygiene, prey foraging and first pellet handling, second pellet handling, brood feeding, and foraging for nest materials. Adult emergence occurred in three synchronized cohorts. Workers began to transition to tasks outside of the nest after 6.3 ± 0.9 days. Younger workers performed hygiene and second pellet processing, mid-aged hornets specialized in prey foraging and first pellet formation, while older workers increasingly foraged for nest materials. Brood feeding was observed by hornets of all ages. Cooperative second pellet processing reduced handling duration by workers compared with first pellets. Of the five potential nest materials deliberately provided, adults most frequently collected hornet nest envelopes and tree bark. Multiple correspondence analysis and clustering confirmed distinct but overlapping task profiles, indicating flexibility instead of a fixed sequence. These findings quantitatively demonstrate age-related division of labor in *V. velutina* similar to that of other social wasps, highlighting behavioral flexibility and cooperative strategies that likely enhance colony efficiency.

## Simple Summary

Hornets are social insects that live in large groups, wherein individuals share many tasks, such as cleaning, prey foraging, and building nests. However, how these activities are divided among workers depending on their age is poorly understood in hornets. In this study, we kept a colony of the invasive hornet *Vespa velutina nigrithorax* in the laboratory and observed the behaviors of newly emerged workers over time. We found that young workers mainly carried out cleaning and food processing inside the nest, middle-aged workers were more involved in prey foraging, and older workers mostly collected nest-building materials. The feeding of larvae was carried out by workers of all ages. We also discovered that food pellets made from prey were processed twice, first outside and then again inside the nest, with several workers cooperating. These results show that workers change their tasks flexibly with age and work together to more efficiently maintain their colony. Understanding these behaviors helps explain the success of this invasive species in new environments.

## 1. Introduction

Maintaining optimal conditions within a colony’s nest is especially crucial for social insects [1,2]. This requires various tasks, such as cleaning, hunting, nest construction, and brood care. Instead of having individuals permanently assigned to a single task, task allocation typically shifts with age, leading to a division of labor among individuals performing different roles [3,4]. This age- or physiology-related task differentiation is known as temporal polyethism [5]. In honey bees (*Apis mellifera*), the most extensively studied eusocial insect, this pattern has been well-studied. During the first 20 days after eclosion, workers primarily engage in cleaning and brood care (nursing). This is followed by 2–3 days of guard duty, after which they transition to external foraging activities, such as collecting water, nectar, and pollen [6]. However, such behavioral transitions are modulated by the demands of the colony. Juvenile hormones (JHs), regulated by internal repressors (IRs), external repressors (ERs), and the allatoregulatory central nervous system, play a key role in determining task allocation [7]. When the brood-to-protein resource ratio is high (i.e., many larvae but little pollen), JH levels increase, thereby accelerating the transition to foraging roles. Conversely, if resources are abundant relative to brood demand, workers tend to continue in their role as nurses.

Temporal polyethism has been reported not only in honey bees but also in many other social insects [8,9]. For example, in rudimentary eusocial wasps of the genus *Ropalidia*, newly emerged workers first perform brood care and nest construction before later shifting to outside tasks, such as foraging, showing a clear temporal polyethism. This sequence of task transitions is closely associated not only with relative age within the colony but also with physiological and chemical traits, such as juvenile hormone and cuticular hydrocarbon profiles [10,11,12,13]. In the swarm-founding epiponine wasp *Polybia occidentalis*, a task organization combining fine-scale age polyethism with pronounced individual task specialization has been described, in which internal and external tasks are partitioned according to worker ages and activity levels and individual behavioral variation is substantial [14,15]. Similarly, in polistine wasps, such as *Mischocyttarus consimilis* (Mischocyttarini), *Polistes canadensis* (Polistini), and *Agelaia pallipes* and *Metapolybia miltoni* (Epiponini), younger workers are mainly involved in brood care and nest maintenance, whereas older workers tend to perform riskier tasks, such as external foraging and nest defense, displaying a typical pattern of temporal polyethism [16,17,18,19]. In contrast, in the highly eusocial wasp *Vespula germanica*, age-related shifts from internal work to foraging do occur though inter-individual variation is high, and a generalist strategy, in which single workers carry out multiple tasks, predominates over strong individual specialization. These studies indicate that patterns of age polyethism and their consistency are diverse among social wasps.

The mechanisms underlying task differentiation in highly eusocial insects, such as ants and honey bees, have been extensively studied. These taxa exhibit highly flexible behavioral regulation systems based on dynamic interactions among colony members and chemical signaling that contribute to colony stability. In contrast, hornets of the genus *Vespa*, which act as apex predators in insect communities, remain poorly studied in this regard [20]. Several species, particularly *V. velutina* and *V. mandarinia*, are notorious predators of honey bees and cause severe ecological and economic damage [21,22,23,24]. *V. velutina nigrithorax* is native to southern China and Southeast Asia; however, since the early 2000s, they have invaded parts of Europe (e.g., France, Spain) and Asia (Korea, Japan), drawing worldwide attention [25,26,27,28,29,30,31]. Heavy predation by *Vespa velutina* on honey bees has led to substantial losses of colonies in apiaries, and predation on diverse pollinators has been reported to affect ecosystem services and biodiversity [26,32,33]. Therefore, understanding not only the impacts of this species but also its behavior and ecology are essential. However, direct observation of worker behavior within the nest is hindered by several factors, including their defensive temperament, concealed nest locations often high in trees, and the presence of a protective nest envelope. Recent studies by Haouzi et al. [34,35] revealed chemical differences associated with worker task in *V. velutina*, including variations in cuticular hydrocarbon profiles and alarm pheromone among workers. However, a knowledge gap remains regarding the presence of age-dependent polyethism in hornets. Like other eusocial insects with relatively large colonies, such as honey bees and ants, *Vespa* are expected to have an age-dependent polyethism. However, whether task frequencies are associated with worker age in *V. velutina* remains poorly understood. Therefore, we investigated the behaviors of individual *V. velutina* hornets by (1) observing a colony under laboratory conditions to identify basic developmental patterns; and (2) examining whether age-related task differentiation and task concurrency occur.

## 2. Materials and Methods

### 2.1. Acquisition of Observation Nest and Its Condition

**Figure 1:**
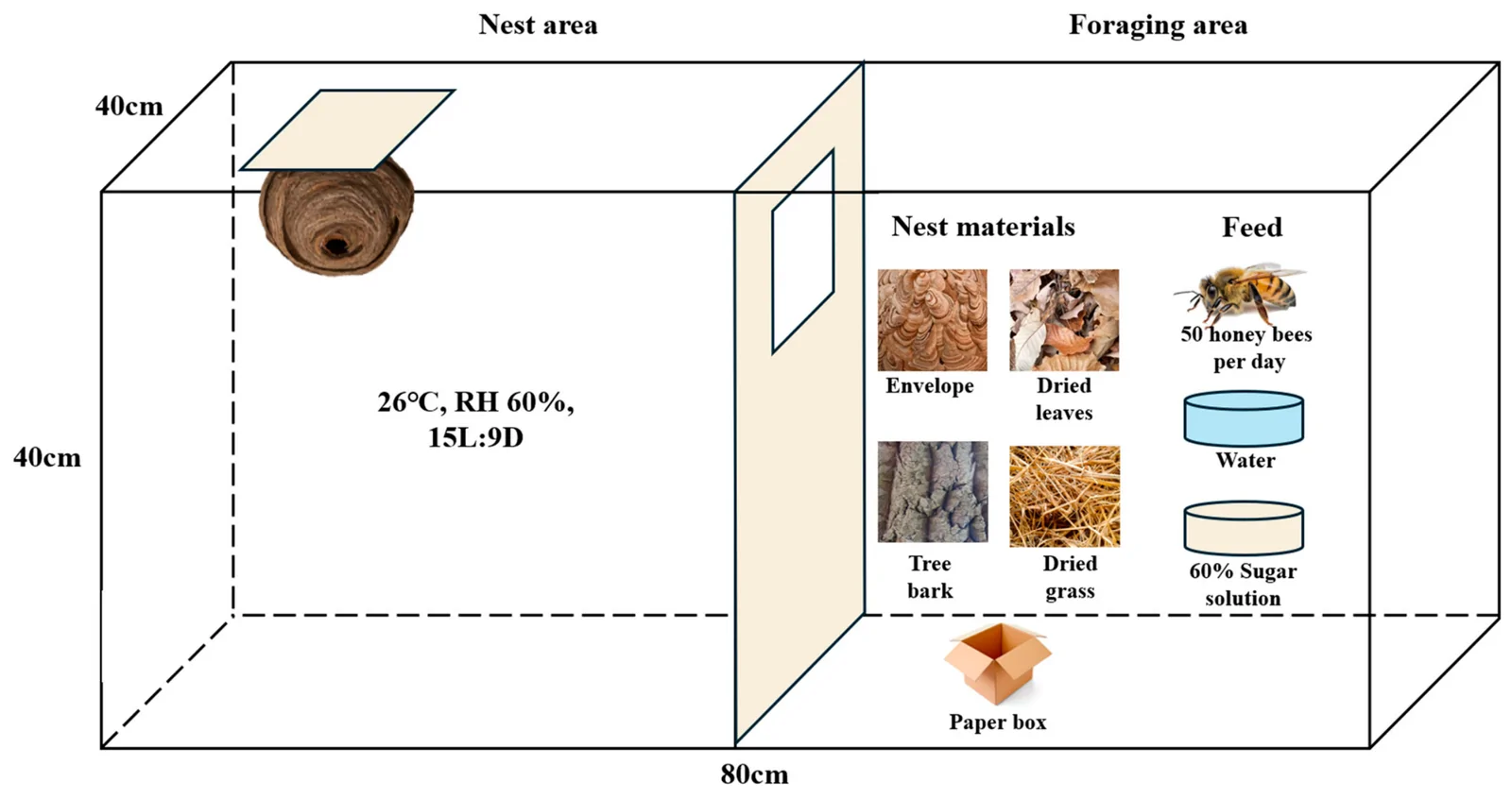
Schematic representation of the rearing conditions in an acrylic cage used for the observation of a *V. velutina* colony. The yellow panel between the nest and the acrylic cage represents a cardboard structure.

A nest of *V. velutina* was collected on 12 June 2024 from Andong, South Korea (36°34′13″ N, 128°43′30″ E). The nest was located beneath a rooftop stairwell of an apartment building in the city center and was collected after sunset (~19:00), when external activity had ceased. At the time of collection, the nest (approximately 10 × 10 cm) consisted of two horizontal combs: the top comb contained 172 cells with 80 pupae, while the second layer comprised 77 cells and 11 pupae. There were 10 adult workers. The collected nest was installed on one side of a transparent acrylic rearing cage (80 × 40 × 40 cm) for indoor observation. A partition wall with a central entrance (10 × 10 cm) was placed in the middle of the cage to separate the space into a nest area and a foraging area. The foraging area was provisioned with a 60% sucrose solution and water, both offered ad libitum via wick feeders. Additionally, fifty recently killed honey bee workers were supplied daily to reinforce the association of this space with the available resources. Workers may encounter potential nest-building substrates that differ in physical structure and processing costs. Therefore, we additionally examined whether workers differed in their use and handling time of experimentally provided nesting materials. Nest-building materials, including used *V. velutina* nest envelope fragments, oak tree bark and dry leaves from around the university, cardboard, and dried grass were also provided in the foraging area. All these materials were dried and disinfected in an oven (60 °C) before being provided. The used nest envelope was included following de Souza et al. [36] to test whether this preference for nest envelope also occurs in the genus *Vespa*. The other four materials, differing in texture, were selected based on our field observations of materials collected naturally by hornets. To minimize contamination from adult excreta or prey remains, a towel was placed on the cage floor beneath the nest and replaced weekly. The colony was maintained under controlled environmental conditions of 26 °C and 60% relative humidity. Light from a nearby window provided natural light–dark cycles (approximately 15L:9D) (Figure 1). Therefore, although the direction of light remained constant, no abnormal worker behavior attributable to phototaxis was observed, as the light intensity was limited and the colony was not exposed to direct sunlight.

### 2.2. Emergence and Foraging Transition

**Figure 2:**
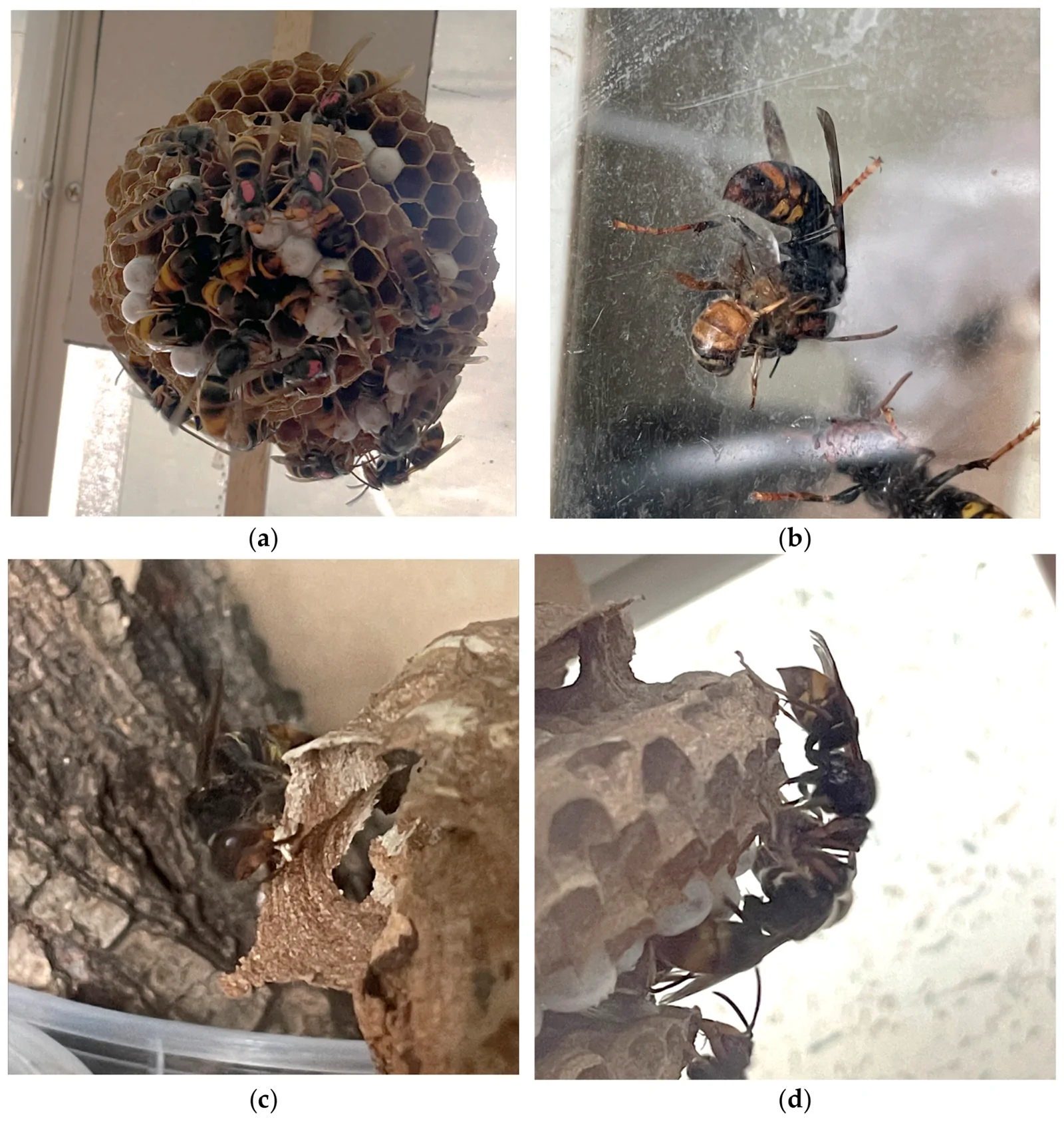
(**a**) A nest installed inside the rearing cage and workers marked with different paint colors according to cohort; (**b**) first pellet handling, in which the head, abdomen, wings, and legs of the prey are removed after prey foraging, leaving only the thorax for further processing; (**c**) a worker collecting used nest envelope from the provided nest-building materials; and (**d**) second pellet handling, where a prey pellet brought back to the nest is further processed and shared with newly emerged internal workers.

Observations were conducted visually from 15 June to 1 August 2024, following a three-day acclimation period to ensure that workers had sufficiently recognized the foraging area. Worker emergence was monitored daily. Throughout the observation period, worker eclosion events occurred at roughly 10-day intervals, and each event was clustered within 3 days; thus, each cluster was defined as a cohort. Newly emerged individuals were marked using paint pens (Posca marker, Mitsubishi Pencil, Tokyo, Japan) of different colors to distinguish three cohorts (first cohort: red; second cohort: white; third cohort: blue) (Figure 2a). The 10 adult workers already present at the beginning of the experiment were not marked because their emergence dates could not be determined. This procedure was performed without anesthetizing the workers. Each wasp was gently restrained by holding the thorax with forceps wrapped in cloth and then marked. Because the colony was at an early developmental stage and previously emerged workers had already been marked, newly emerged workers could be readily distinguished by the absence of paint marks. To prevent stinging and escape, the procedure was conducted inside a meshed tent while the handler wore protective hornet clothing and rubber gloves. After marking, the workers were immediately returned to the rearing cage. To assess foraging transitions, behavioral surveys were conducted five times per day (the smallest interval between observations was 1 h). Because all cohorts completed emergence within three days, we estimated the time required for transition to foraging by using the midpoint date of each cohort as a reference. For each observation, the number of workers present in the foraging area, the cohort of each worker observed, and their behaviors at the time of observation were recorded.

### 2.3. Temporal Labor Division

Under indoor rearing conditions, the temporal task performance of *V. velutina* workers was examined. Due to the clustered timings of emergence within each cohort, workers were categorized according to cohort and their age in days after eclosion. Age was divided into six groups at 5-day intervals. Tasks were categorized into five visually distinguishable types: hygiene, prey foraging and first pellet handling (Figure 2b), second pellet handling (Figure 2d), brood feeding, and foraging for nest materials (Table 1, Figure 2c). Each task was determined based on the worker’s behavior at the time of observation.

### 2.4. Resource Collection and Handling

Resource collection and processing were assessed by dividing the time required for pellet handling into two phases, the external (first pellet handling) and internal (second pellet handling) stages, as the process occurs in two distinct steps. Since multiple individuals participated in the internal pellet handling within the nest, the number of workers involved in sharing each pellet handling task was also recorded (*n* = 30). In addition, the preference among the five nest-building materials was evaluated, along with the time required to collect each of them (*n* = 47).

### 2.5. Data Analysis

In this study, three analyses were conducted to examine the distribution and structural relationships of task types across age categories (groups). First, multiple correspondence analysis (MCA) was performed to explore the association between age groups and task types and to visualize their separation along major dimensions. Second, the relative frequency distribution of behaviors performed by each group was quantitatively assessed using bar plots to identify age groups that predominantly performed specific tasks. Third, to numerically compare behavioral similarity between groups a Euclidean distance matrix was computed and visualized using hierarchical clustering to reveal the distance structure and clustering patterns between groups.

To compare the time required for the first and second pellet handling, a Mann–Whitney U test was performed. Collection durations for different nest-building materials were analyzed using the Kruskal–Wallis test, and if significant differences were found, Dunn’s test with Bonferroni correction was conducted as a post hoc analysis. A significance level of 0.05 was used for all tests. All statistical analyses were performed using R (Version 1.4.1106; RStudio, PBC, Boston, MA, USA).

## 3. Results

### 3.1. Cohort Emergence

**Figure 3:**
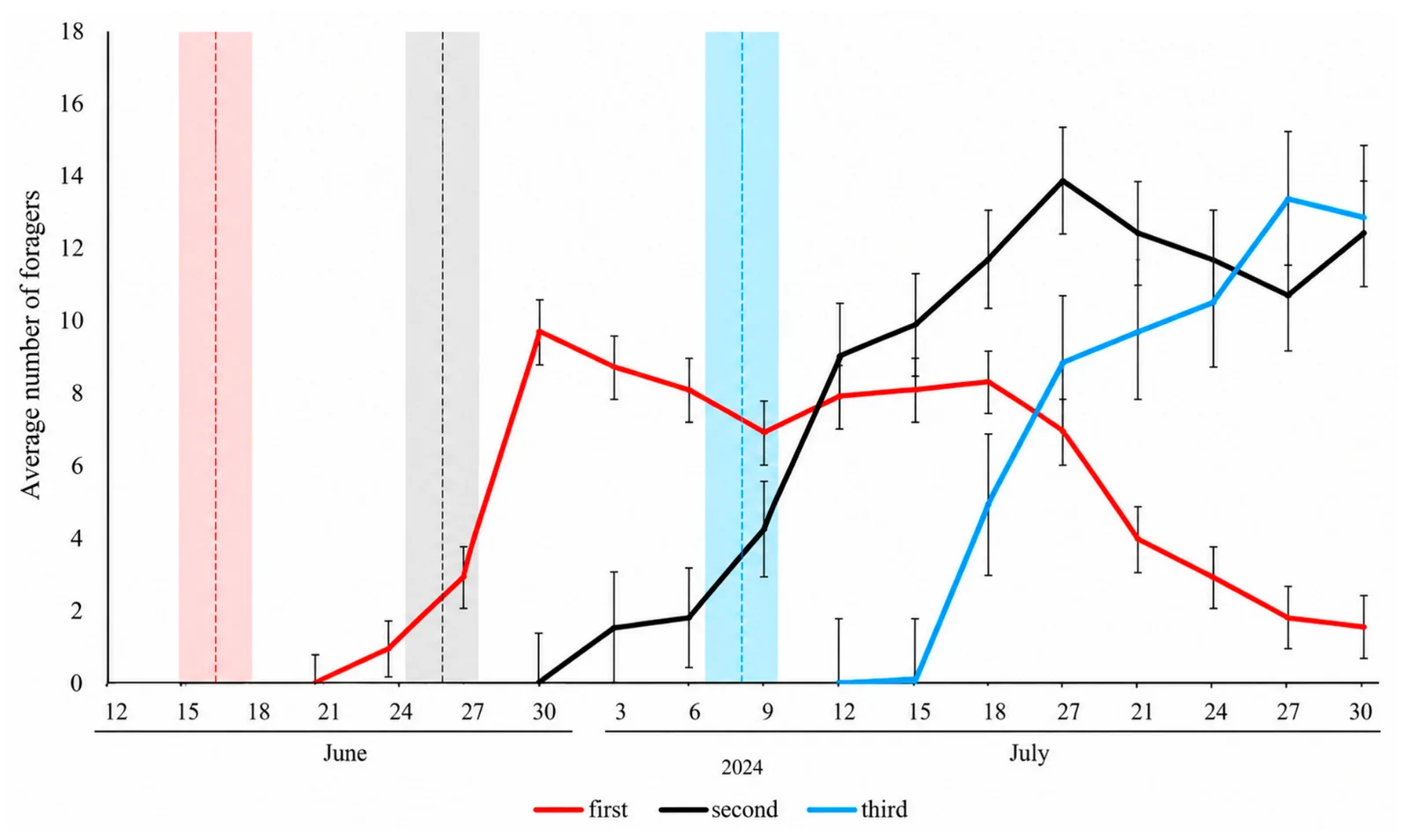
The average number of foraging workers observed in the foraging area. Each color shade indicates the emergence period of each cohort (first cohort: red, second cohort: black, third cohort: blue) and dotted line is median date. Error bars represent standard error.

Three distinct cohorts emerged during the observation period, with eclosion within each cohort occurring in a concentrated window of three days. A total of 78 adult workers emerged during the study (e.g., first cohort—red (*n* = 40), second cohort—white (*n* = 21), third cohort—blue (*n* = 17)). On average, 29.3 ± 9.5 (SD) workers emerged per cohort, and the interval between cohorts was 11 ± 4 (SD) days (Figure 3). Because the colony was maintained under controlled laboratory rearing conditions, no early mortality was observed during the experimental period. For quantification of behaviors, hornets were divided into six 5-day age groups. Newly emerged workers were primarily observed between the first and second combs. During this period, they relied on larval saliva and trophallaxis among adults for food. Nutrient exchange among adults was initiated when a newly emerged worker stimulated the mouthparts of a donor individual with its mandibles, prompting the donor to extend its proboscis and transfer food. These exchanges typically lasted from 2 to 3 s up to 10 s. Similarly, when stimulating larval mouthparts with their mandibles, workers consumed the regurgitated larval saliva. However, even after transitioning to foraging-related tasks, workers appeared to rely on trophallaxis with larvae for carbohydrate intake. Consumption of the sugar solution provided in the foraging area was rarely observed and was not associated with a specific age group. No development of drone or queen cells or brood was observed during the study.

### 3.2. Foraging Transition and Task Differentiation

**Figure 4:**
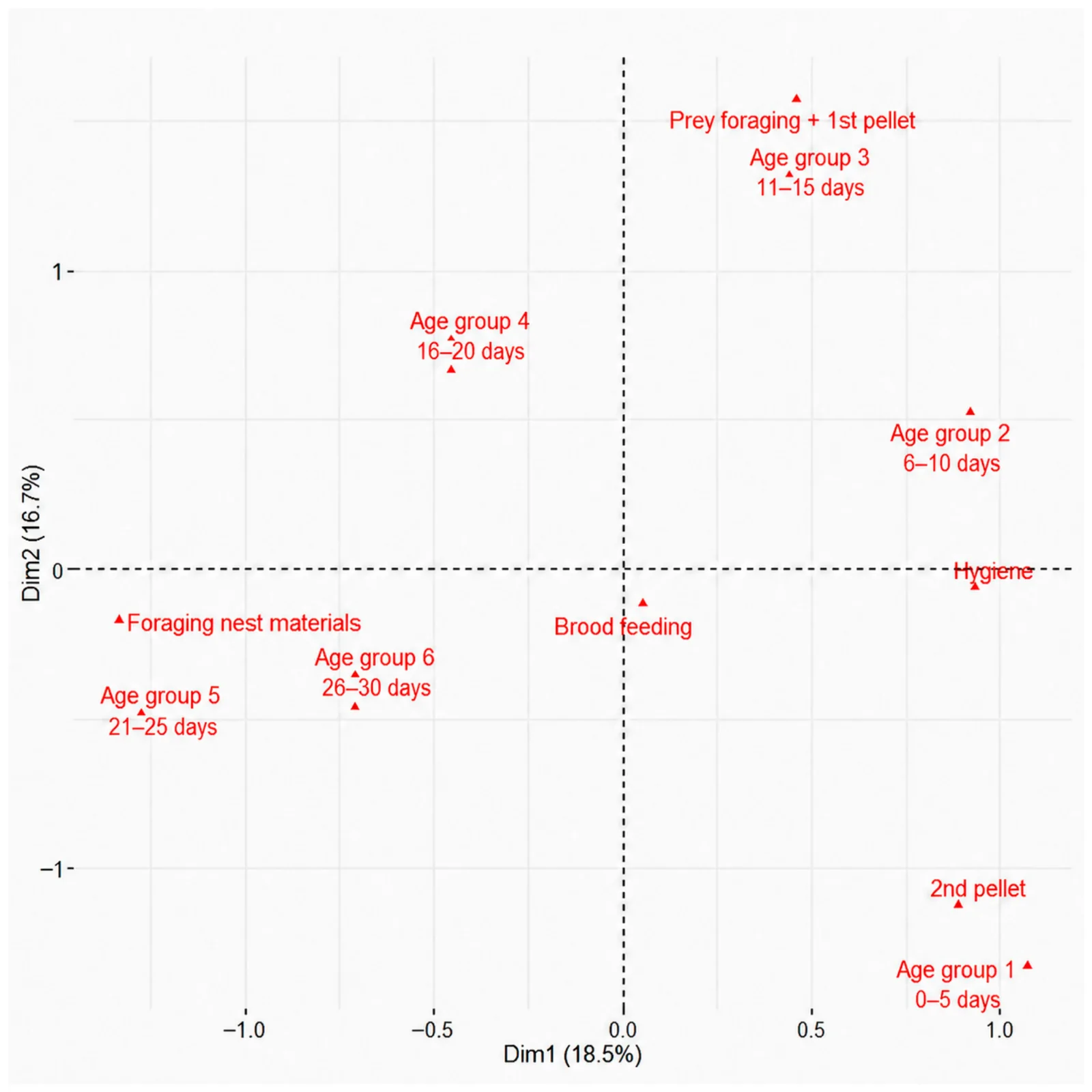
Multiple correspondence analysis (MCA) biplot illustrating associations between age groups (Groups 1–6) and task categories in *V. velutina*. Each point represents a categorical variable, and spatial proximity indicates behavioral similarity. Dimension 1 (18.6%) reflects the transition from internal to external tasks, while Dimension 2 (16.7%) captures mid-phase roles, such as prey foraging and pellet processing. Full dimension statistics are provided in Table A1.

On average, workers began to transition to external foraging 6.3 ± 0.9 (SD) days after emergence (Figure 3). The results from the multiple correspondence analysis (MCA) revealed that Dimension 1 (Dim. 1) and Dimension 2 (Dim. 2) accounted for 18.6% and 16.7% of the total variance, respectively, explaining a combined 35.3% of the dataset. Tasks such as hygiene (contribution = 6.35%, cos^2^ = 0.121) and second pellet handling (contribution = 9.64%, cos^2^ = 0.202) were primarily performed by younger workers in Groups 1 and 2 (0–10 days of age), contributing strongly to Dim. 1. In contrast, foraging for nest materials was located on the negative axis of Dim. 1 and showed the highest explanatory power (contribution = 31.5%, cos^2^ = 0.748), indicating that this task was most closely associated with Groups 5 and 6 (21–30 days of age) and was frequently performed later in life. Prey foraging and first pellet handling (contribution = 32.16%, cos^2^ = 0.748) were concentrated in Groups 2 to 4 (6–20 days of age). Brood feeding was observed across all age groups with relatively uniform distribution (Figure 4). The approximate lifespan of workers observed in this study was 30–45 days. After reaching the final age category (Group 6), most workers gradually died either inside the nest or in the foraging area, with little or no task activity observed. Thus, observations beyond Group 6 were excluded from the analysis.

**Figure 5:**
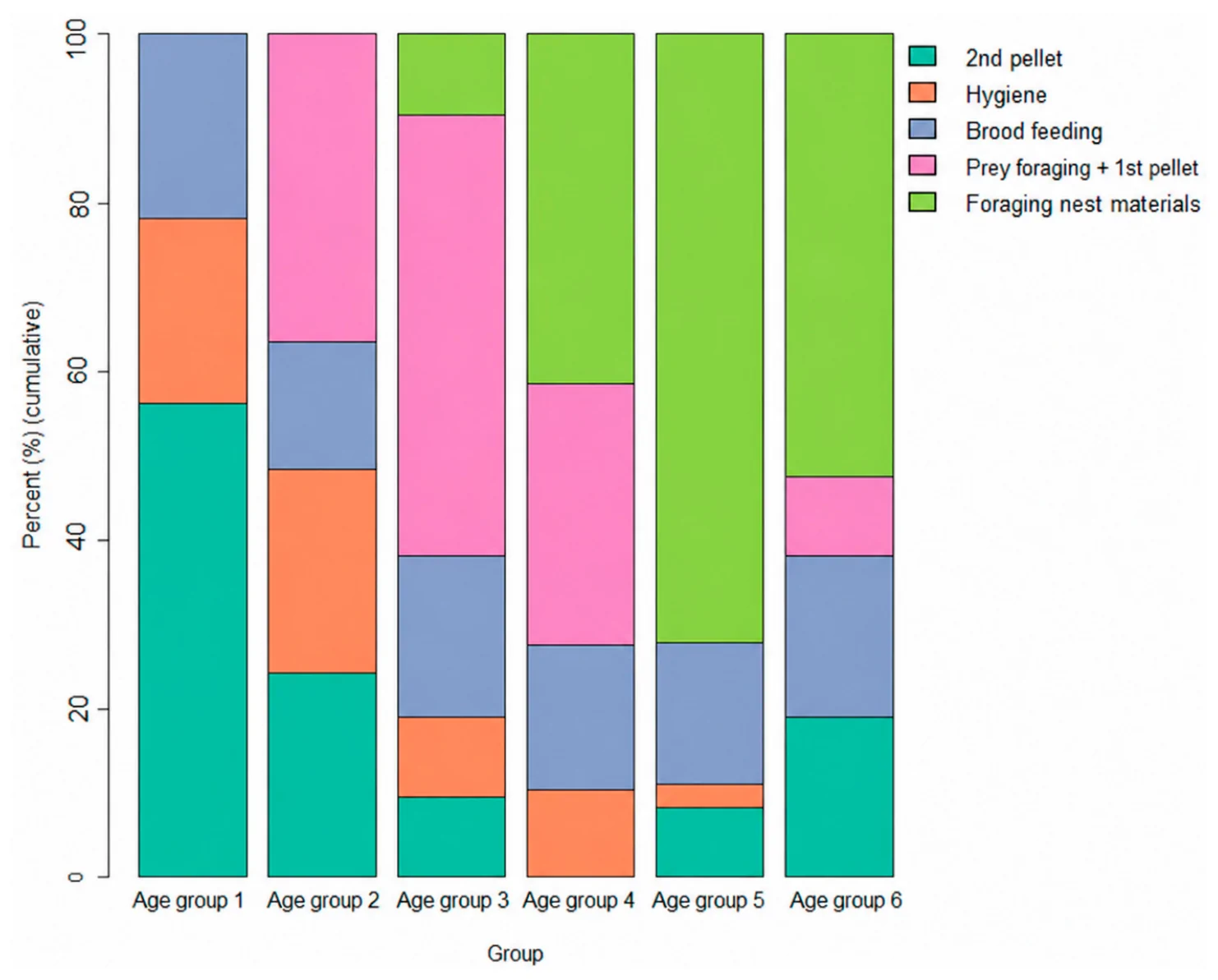
Relative frequency (%) of five task types performed by *V. velutina* workers across six age groups (all cohort combined). Blocks within each age group represent the proportions of individuals engaged in each task. Early-stage groups (Groups 1–2) mainly performed internal tasks such as second pellet handling and hygiene, whereas mid-age groups (Groups 3–4) showed a peak in prey foraging and first pellet handling. Foraging for nest materials became dominant in late-stage groups (Groups 5–6), indicating a shift from prey foraging toward nest material collection with age.

An analysis of task frequencies across age groups supported these patterns. While second pellet handling occurred in all groups, it was primarily concentrated early in life, as was hygiene. Prey foraging and first pellet handling was not observed until at least six days post eclosion, peaking in Group 3 and then declining in older groups. In contrast, foraging for nest materials notably increased in Group 4 (16–20-day-old workers) and continued into the latest age group (Figure 5).

**Figure 6:**
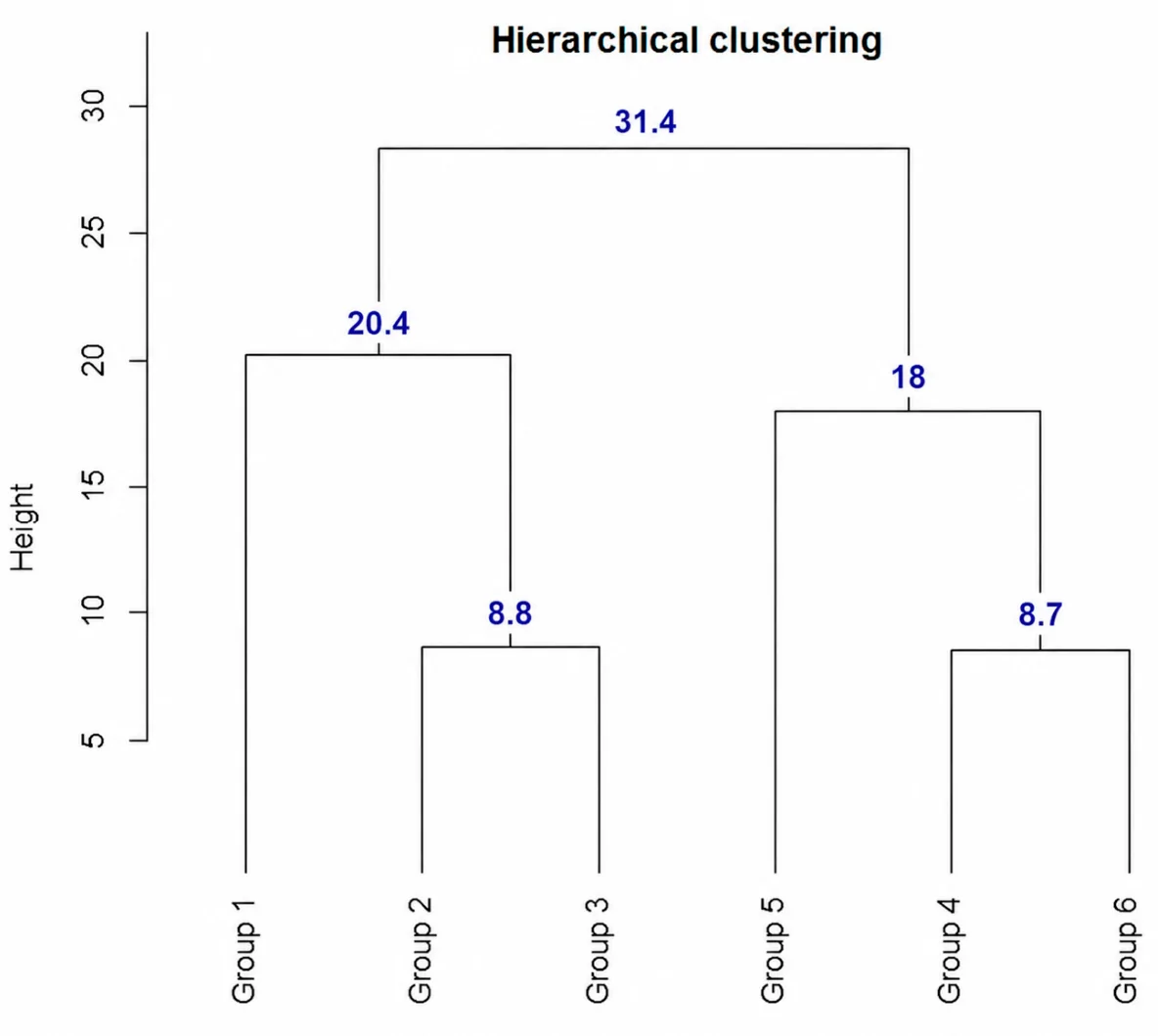
Hierarchical clustering dendrogram of *V. velutina* worker age groups based on task performance profiles. Clustering was performed using Ward’s method on Euclidean distances calculated from the group-by-task frequency matrix. The analysis revealed three major clusters with (i) Group 1, (ii) Groups 2 and 3, and (iii) Groups 4–6, suggesting clear age-related shifts in task allocation. The linkage heights indicate the dissimilarity between group profiles, with shorter heights reflecting more similar behavioral patterns.

Euclidean distance analyses of group centroids showed that Groups 1, 2, and 3 clustered together, with Groups 2 and 3—the initial external foragers—showing the shortest distance between them (Euclidean distance: 8.8), indicating highly similar behavioral patterns. The older groups (groups 4–6) also exhibited a relatively consistent pattern of external task engagement. Younger groups were characterized by a focus on internal tasks, such as hygiene and pellet processing, whereas older groups specialized in collecting nest-building materials, showing a clear behavioral shift across the worker lifespan (Figure 6).

### 3.3. Pellet Handling

**Figure 7:**
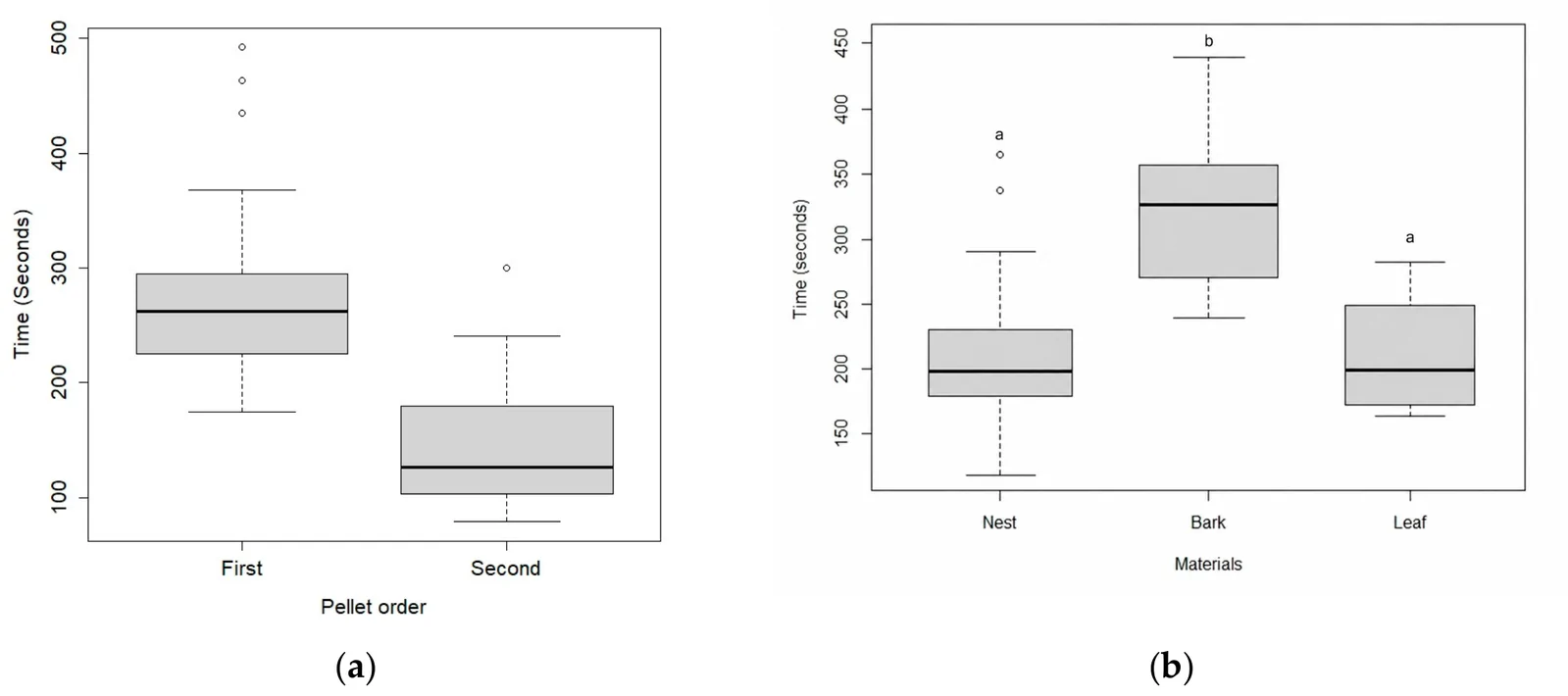
(**a**) Duration of first pellet (*n* = 30) and second pellet (*n* = 30) handling by *V. velutina* workers. Boxes represent the interquartile range (IQR), horizontal lines indicate medians, whiskers extend to the most extreme values within 1.5 × IQR, and points indicate outliers. The second pellet, formed cooperatively inside the nest, took significantly less time than the first (U = 842, *p* < 0.001). (**b**) Time required for bouts of foraging for nest materials by *V. velutina* workers. The sample sizes represent the number of collection bouts observed for each material: used nest envelope (n = 29), oak tree bark (n = 14), and dried leaves (n = 4). Duration was measured from the start of scraping the material until the worker completed pellet formation. Bark required the longest handling time (χ^2^ = 21.7, df = 2, *p* < 0.001).

Pellet handling occurred not only externally but also within the nest. The first (external) pellet handling took longer (275 ± 8 s; mean ± SD) than the second (internal) pellet handling (141 ± 51 s) (U = 842, *p* < 0.001; (Figure 7a)). During the second pellet handling, an average of 3.1 ± 0.5 workers cooperatively processed each pellet (*n* = 30), and solitary processing was never observed. In contrast to nest material, prey pellets elicited an immediate response from nest workers which rapidly approached the returning forager and participated in pellet transfer and subsequent processing. Each completed pellet was typically fed to an average of 4.2 ± 1.3 larvae, though the number varied between two and seven depending on larval size. Although the exact quantity of food was not measured, workers generally detached a portion approximately twice the size of the larva’s head and placed it at the ventral side of the larval mouthparts for feeding.

### 3.4. Nest Material Handling

Among the five nest materials provided, only three selected by the workers: used nest envelope (*n* = 29), oak tree bark (*n* = 14), and dried leaves (*n* = 4) in order of preference. The time required for collecting and processing each material was 209 ± 56 s (mean ± SD) for used nest envelope, 324 ± 57 s for oak tree bark, and 210 ± 45 s for dried leaves. Handling times differed significantly among the three materials (χ^2^ = 21.7, df = 2, *p* < 0.001) (Figure 7b). Oak tree bark required the longest handling time.

## 4. Discussion

In this study, we quantitatively confirmed the existence of temporal polyethism in a *V. velutina* colony under controlled laboratory conditions. Hornet behaviors followed a progression from internal tasks, such as hygiene and brood care, that declined in frequency over time as hornets transitioned to external tasks, with prey foraging generally occurring more frequently than collection of nest-building materials. However, all external tasks were performed concurrently at the colony level. This behavioral sequence was consistently observed across all three cohorts.

In social Hymenoptera, juvenile hormones (JHs) function as endocrine signals that promote behavioral maturation. In *Polybia occidentalis*, treatment of workers with the JH analog methoprene advances age polyethism and accelerates the shift to foraging [14]. Moreover, experimental manipulation of JHs concurrently alters ovarian activation and cuticular hydrocarbon (CHC) profiles, indicating that task allocation, physiological state, and chemical signaling are integratively regulated along the JH axis [37]. However, task transitions appear to be regulated not only by hormonal control but also more simply by external stimuli, reflecting differences in colony sizes, ecological niches, and evolutionary levels of sociality [38,39].

In *V. orientalis*, a subterranean-nesting hornet native to Southwest Asia, the Mediterranean, and parts of northern Africa, individual task specialization, based both on activity frequency and task type, has been observed regardless of temporal age [40]. Individual specialization refers to a behavioral pattern in which a given worker persistently and intensively performs a specific task, instead of transitioning through different tasks over time. However, such specialization may vary depending on the individual’s characteristics and colony’s needs. This phenomenon also has been documented in *Polistes*, a representative model taxon of primitively eusocial insects, in which both polyphenism and polyethism are often ambiguous [41,42]. In *Mischocyttarus mastigophorus*, workers with more developed dorsal longitudinal muscles have been reported to engage more frequently in tasks that require flight, such as external foraging, supporting the concept of individual task specialization [39]. In contrast, species of the genus *Vespula*, including *Vespula germanica*, often exhibit characteristics of both temporal polyethism and individual specialization [43,44,45,46,47]. Although patterns vary depending on sample size and colony, workers are generally reported to follow a task sequence of internal tasks → foraging for nest materials → foraging for carbohydrates → prey foraging. However, following nest material collection, workers often perform both carbohydrate foraging and hunting concurrently. This flexible form of polyethism, in which workers perform multiple external tasks within the same age group after transitioning to foraging roles, aligns with the findings of the present study albeit with a different sequence of behaviors.

The predominance of nest material collection during the later stages of the workers’ lifespan may be associated with age-related changes in physical condition. Repeated performance of mechanically demanding tasks can result in cumulative mandible wear, which may influence task allocation during the later stages of workers’ lives [48,49,50]. When workers are in optimal physical condition, they can efficiently perform a wide range of tasks, whereas tasks that result in significant physical wear tend to be performed during the later life stages. This age-related allocation of tasks may contribute to maintaining colony-level efficiency by matching task performance with changes in worker condition across the lifespan [51,52,53].These findings suggest that the task transitions observed in this study do not follow a rigid, fixed sequence. Instead, they appear to be regulated by a flexible mechanism in which task allocation is modulated according to both colony needs and individual worker conditions within a general task transition flow.

*V. velutina* is a generalist predator that feeds on a wide range of arthropods, including Hymenoptera, Diptera, Hemiptera, and Araneae. However, honey bees are its preferred prey [33,54,55,56]. *V. velutina* typically captures honey bees around beehive entrances, after which they land on nearby branches and remove the head, abdomen, and wings before masticating the thorax with saliva to form a pellet which is then carried back to the nest [57,58]. The time required for this process has been previously reported as approximately 270 ± 55 s, which closely matches the 275 ± 79 s observed in this study [57]. Notably, this study also confirmed that further pellet processing occurs within the nest. Although such internal behavior is generally impossible to observe in the field due to the spatial separation between the nest and foraging site and the opaque nest envelope, indoor observations revealed that several workers cooperatively processed each pellet for an additional two to three min. The final pellets delivered to the larvae were more finely masticated than the initially prepared external pellets, suggesting that secondary processing further reduced the size and altered the consistency of prey material before larval feeding.

Furthermore, when a forager returned to the nest with a prey pellet, nearby internal workers immediately gathered and contributed to pellet processing. In contrast, when nest-building materials were brought in, no such response was observed. These differences suggest that workers may recognize and respond to task types based on cues, such as odor or behavior. In a previous study of *V. velutina*, workers that engaged in animal (prey) pellet foraging, as well as those involved in nest building, differed in the composition of their cuticular hydrocarbons (CHCs), including alkenes and branched alkanes [34]. Differences were also observed in the alarm pheromone composition of the venom glands between workers performing these two roles, suggesting that such chemical variation may be associated with behavior, the materials handled, and the frequency of outdoor activities [35].

Although water and a 60% sucrose solution were provided in the foraging area, few individuals were observed consuming these resources. Carbohydrate intake by both workers and the queen was derived primarily from larval saliva. In addition to carbohydrates, larval saliva contains various amino acids, such as proline, which contribute not only to adult nutrition but also to the sexual maturation of reproductives [59,60,61]. It has also been reported that larval saliva serves as a form of reward that enhances workers’ foraging motivation [62].

**Figure A1:**
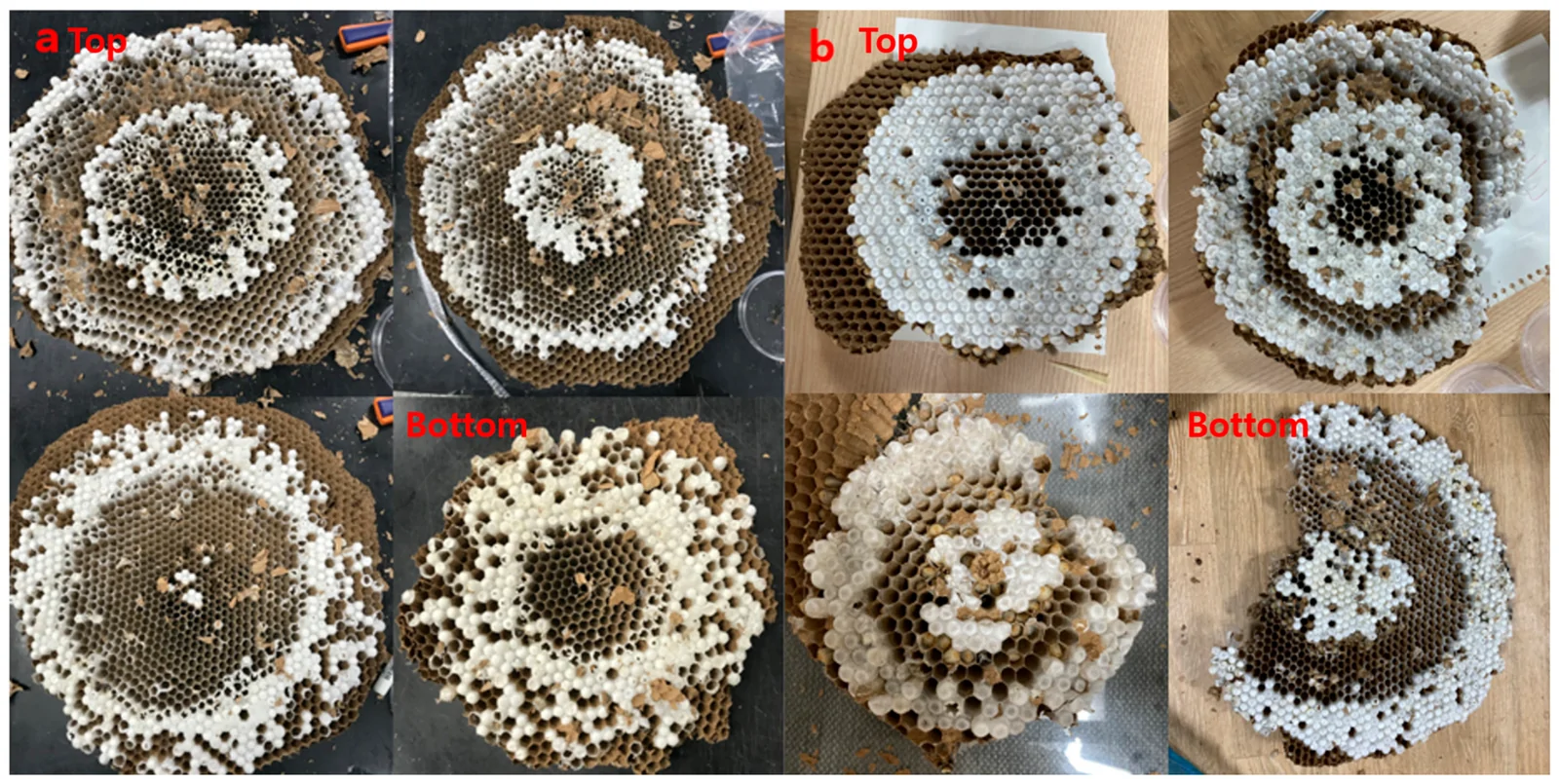
Alternating spatially segregated sealed-brood patterns in autumn *V. velutina* nest combs: (**a**) nest collected in September 2023, Gunwi (36°16′12.21″ N, 128°28′51.23″ E); and (**b**) nest collected in September 2023, Andong (36°29′49.59″ N, 128°36′01.00″ E).

In this study, all individuals within a single cohort emerged within a three-day period. When one cohort occupied the central portion of the nest, the subsequent cohort was arranged in a surrounding layer, and the next cohort was again located centrally. This spatial pattern also has been observed in mature nests during the peak of the autumn season (personal observation, Figure A1), suggesting that the queen’s oviposition is not continuous or sporadic but rather concentrated in both timing and location once a set of cells is completed. However, such patterns may vary depending on the cohort interval and the availability of nest cells [63]. In the present study, the interval between cohorts was approximately 11 ± 4 days, although this may be influenced by internal factors, such as temperature, nutrition, and colony structure, as well as external factors, including ambient temperature, seasonal variation, predators, and disease-related stress [64].

Because the observations were conducted under laboratory conditions, the overall task intensity was notably low, and all necessary resources were continuously provided ad libitum. Therefore, the behavioral patterns observed may differ from those under field conditions. Furthermore, as the study was conducted on a single colony, potential colony-specific variations are possible. Although a consistent task flow was observed across all three cohorts in this study, these results were obtained from a single colony and should therefore be interpreted with caution. In social insects, between-colony variation is an important consideration as the intensity of behavior and the degree of responsiveness to external stimuli can differ substantially between colonies [65,66]. This is consistent with the idea that selection pressures, such as predation risk, resource availability, and environmental disturbance, often act at the colony level rather than solely at the individual level, potentially giving rise to colony-specific behavioral tendencies or personalities [67]. Empirical evidence for such variation has been reported in several eusocial insects. In honey bees for example, colonies differ consistently in defensive behavior, foraging activity, and hygienic behavior, and some of these traits are linked to productivity and overwinter survival [68]. In the harvester ant *Pogonomyrmex barbatus*, colony-specific differences have also been reported in the relationship between foraging activity and humidity [69]. Likewise, in *Vespula wasps*, colony-level variations in aggression toward external stimuli have been documented [70]. Therefore, the extent to which the patterns observed in this single colony are representative of other *V. velutina* colonies remains uncertain. Nevertheless, because our observations were conducted under controlled laboratory conditions, environmental variation was minimized, allowing us to provide a detailed baseline description of age-dependent task allocation and behavioral transitions in *V. velutina*. Future studies should test the generality of these patterns using multiple colonies under both laboratory and field conditions, where additional ecological disturbances and environmental heterogeneity can be incorporated.

We quantified the age-based task differentiation and transition flow of worker *V. velutina* within their nest. Future research should aim to investigate the relationship between behavioral transitions and physiological markers, such as juvenile hormones and vitellogenins, which are known to regulate task shifts. Additionally, studies examining how the composition of cuticular hydrocarbons (CHCs) changes with age and task allocation are warranted. These physiological mechanisms and social chemical signals likely mediate individual interactions and support flexible polyethism, which may together serve as a key adaptive strategy that enables *V. velutina* to rapidly establish and maintain colonies in novel environments.

## Figures and Tables

**Table 1:** The tasks observed to assess the division of labor and the criteria used for evaluation.

Tasks	Criteria
Hygiene	Removing dead larvae and discarding them outside the nest, and cleaning debris on the comb or envelope fragments
Prey foraging and first pellet handling	Prey foraging: grasping honey bees and removing the head; First pellet handling: removing the gaster and appendages and rolling the pellet outside the nest
Second pellet handling	Taking the first pellet into the nest, then rolling the pellet with other workers for feeding inside the nest
Brood feeding	Feeding small pieces of the second pellet to larvae
Foraging for nest materials	Collecting nesting materials in the foraging area

## Data Availability

The data/findings of the study are available upon request to the corresponding authors.

## Appendix A

**Table A1:** Summary statistics of MCA for task categories and age groups. Dim = dimension; Coord = coordinate; Cos^2^ = squared cosine (quality of representation); Contribution = contribution to the dimension (%); v-test = standardized contribution of category to the axis.

Category	Dim.	Coord	Contribution (%)	Cos^2^	v-Test
Group 1	1	1.069	12.74	0.261	6.684
	2	−1.325	21.69	0.401	−8.285
Group 2	1	0.92	9.73	0.201	5.863
	2	0.522	3.48	0.065	3.328
Group 3	1	0.443	1.44	0.027	2.161
	2	1.32	14.12	0.242	6.435
Group 4	1	−0.453	2.07	0.042	−2.668
	2	0.763	6.52	0.118	4.494
Group 5	1	−1.274	20.35	0.43	−8.571
	2	−0.478	3.18	0.061	−3.219
Group 6	1	−0.709	3.67	0.07	−3.456
	2	−0.355	1.02	0.018	−1.730
Second pellet	1	0.889	9.64	0.202	5.877
	2	−1.123	17.03	0.322	−7.420
Brood feeding	1	0.054	0.03	0.001	0.329
	2	−0.119	0.17	0.003	−0.727
Foraging for materials	1	−1.332	31.5	0.748	−11.306
	2	−0.176	0.61	0.013	−1.495
Prey foraging + first pellet	1	0.458	2.48	0.052	2.972
	2	1.565	32.16	0.604	10.161
Hygiene	1	0.932	6.35	0.121	4.544
	2	−0.061	0.03	0.001	−0.297
